# Specific challenges of researching stress in the context of quiet political repression

**DOI:** 10.1016/j.cpnec.2024.100248

**Published:** 2024-07-01

**Authors:** Ruth Marheinecke, Ann-Christin Winter, Bernhard Strauss, Veronika Engert

**Affiliations:** a Institute for Psychosocial Medicine, Psychotherapy and Psychooncology, Jena University Hospital, Friedrich-Schiller University, Jena, Germany; b Department of Clinical Psychology, Justus-Liebig University, Gießen, Germany; c German Center for Mental Health (DZPG), Partner Site Halle-Jena-Magdeburg, Germany; d Center for Intervention and Research on Adaptive and Maladaptive Brain Circuits Underlying Mental Health (C-I-R-C), Halle-Jena-Magdeburg, Germany

**Keywords:** Chronic stress, GDR, Political repression, Quiet or soft repression, Study design, Trauma

## Abstract

Political repression beneath the threshold of criminal prosecution is a phenomenon of past and present, predominantly authoritarian, regimes. This so-called *quiet* repression includes measures such as the limitation of freedom of speech, surveillance of (perceived) political opponents, or the spreading of rumors to socially isolate targets. Such experiences of chronic stress show significant psychological and physiological health consequences in affected individuals. However, societal awareness of quiet repression measures remains limited, hindering victims' access to support and complicating healthcare interventions. In the current paper, we present the design of a study conducted with individuals who endured quiet repression measures in the former German Democratic Republic (GDR), a socialist state closely aligned with the former Soviet Union. We discuss the challenges encountered over the course of the study, and present the solutions found. Although every study population has their unique challenges and needs, we wish to inform future sensitive research within the realm of quiet political repression. Given the limited understanding of the phenomenon, there is a pressing need for further investigation aiming to improve acceptance and care for past and future victims.

## Highlights

• Quiet political repression includes measures such as the limitation of freedom of speech, surveillance, or the spreading of rumors to socially isolate (perceived) political opponents.
• Quiet political repression induces chronic psychosocial stress, affecting mental and physical health.
• Challenges in studying quiet repression include recruitment difficulties and heterogeneity of experiences.
• Stress measurements need to be adapted to the needs of the population investigated.
• Given the risk of reactivating traumatic memories, psychological support is crucial for participants.

## 1 Introduction

In times of climate change, war and forced migration, research on how our social environment influences our mental and physical health is pivotal. In the past, important research has focused on the consequences of experiencing trauma, such as war combat, sexual and physical abuse, or natural catastrophes, often in the context of Posttraumatic Stress Disorder e.g. Refs. [[1], [2], [3], [4], [5]]. One area that still remains understudied are experiences of covert repression outside the range of classical trauma or physical violence. Internationally, this type of repression is often referred to as soft repression e.g. Ref. [6]. To stay true to the established wording in the context of covert repression in the former German Democratic Republic (GDR; 1949–1990), we here use the term *quiet* repression.

Quiet repression techniques still play an active and increasingly salient role predominantly in authoritarian regimes today e.g. Refs. [[7], [8], [9], [10]]. As studies in Hong Kong, Russia or Nicaragua show, state regimes use measures that are less visible than overt physical violence, including, for example, surveillance, the limitation of freedom of speech, or the creation of failures in private or professional life to avoid raising public attention for ongoing violations of human rights. By these means, political opponents can be deprived of valuable resources, such as time, money, security or allies [[7], [8], [9], [10], [11]]. Quiet repression aims to create ongoing feelings of uncontrollability, social threat, and uncertainty – which form the core elements of psychosocial stress [12].

One prominent example of quiet repression were specific practices used in the former GDR, an authoritarian regime in East Germany, closely linked to the former Soviet Union. Routinely applied, practices included wiretapping, spreading of rumors, or provoking failures in professional and social domains, all of which aimed to systematically undermine an individual's psychosocial integrity by inducing anxiety, panic, social isolation and confusion [13]. Like the “classical” instances of trauma, noncriminal repression was shown to lead to severe mental and physical health consequences for the victims, even many years after their initial experiences [14,15].

Despite these significant health consequences, societal awareness for the impact of quiet repression is low. Accordingly, accessing rehabilitative state funds remains a difficult undertaking for victims of repression in the GDR, and health care professionals oftentimes lack the understanding to properly treat affected victims [16,17]. It is little surprising that victims are unsatisfied with the public handling of the issue. Quite possibly, the prevailing lack of acknowledgement for the health consequences of quiet repression is rooted, at least partially, in the insufficient knowledge of the actual mechanisms behind these health consequences. In contrast to other forms of repression (e.g. imprisonment, torture), quiet repression is not easy to measure, operationalize or grasp in general. To this end, it is vital to more closely understand not only the form of repression itself, but also the physiological mechanisms through which it impacts health. However, and this leads to the specific focus of the current methodological paper, it poses a special challenge to access and study victims of quiet repression. While some researchers have worked directly in regions of crises e.g. Ref. [18], we studied a sample of individuals who experienced quiet repression in the former GDR more than 30 years ago.

We here expand on the importance of investigating the psychobiological consequences of quiet repression [12], while highlighting the challenges therein. Beginning with an overview of the stress response and the classification of quiet repression as a prototypic chronic psychosocial stressor, we present the most frequently applied research methods to capture stress. We then outline the setting of a study conducted in victims of quiet political repression in the former GDR. Overall, our aim is to share insights into the challenges of assessing psychosocial stress and its consequences in the context of political repression, and illustrate the solutions found in the context of our specific study.

## 2 Psychosocial stress and its consequences

The mechanisms used in quiet political repression are rooted in the induction of severe and chronic psychosocial stress in target individuals [12]. Stress is commonly defined as an organism's response to a perceived threat to its homeostasis (internal equilibrium) through either internal or external adverse forces termed stressors [19]. Homeostasis is then re-established by various behavioral and physiological responses. The principal end-effectors of the physiological stress response are cortisol, released by the hypothalamic–pituitary–adrenal (HPA) axis, and epinephrine and norepinephrine, released by the sympathetic branch of the autonomic nervous system (ANS). Chronic stress has been shown to promote pathophysiological changes, leading to mental and physical health impairments, including depression, anxiety, metabolic, cardiovascular, and autoimmune disorders e.g. Refs. [[19], [20], [21]].

Not every stressor induces the same response in every individual. Contextual and individual factors such as societal norms, past experiences, coping mechanisms and individual appraisals influence individual resources [[22], [23], [24], [25]]. Next to contextual and individual factors, there are stressor-inherent aspects that have an amplifying impact on the stress response. Thus, if stressors are persistent, uncontrollable, unpredictable, involve a threat to the self, and trigger shame, dysregulation of the stress and immune systems is more likely to occur, leading to an elevated risk for the development of mental and physical illnesses e.g. Refs. [[26], [27], [28], [29]].

### 2.1 Political repression as a psychosocial stressor

The above described core mechanisms of psychosocial stress are inherent to the covert repression techniques of the former authoritarian regime of the GDR [12]. Aim of these techniques was to silence (perceived) political opponents by causing life crises, inflicting psychological strain, and undermining self-worth [13], thereby fostering fear, panic, and confusion, and targeting the psychosocial integrity of victims. The utilized repression measures were personalized and anonymous, implemented by a broad network of unofficial employees of the secret police (MfS; Ministry of State Security), as well as employers, teachers, and the police itself [13,[30], [31], [32]]. Studies indicate that the chronic psychosocial stress experienced in the context of quiet, non-criminal repression can lead to comparable, long-lasting impairments in mental and physical health as the experience of a “classical” trauma such as political imprisonment [14,15,33].

Noticing the similarities of quiet repression with severe chronic psychosocial stress underlines the importance of extending investigations into the consequences of quiet repression to the function of stress-regulatory systems. In light of the likely vulnerability of former victims, however, it is imperative to adapt the existing methods in stress research to the specific needs and challenges of the population.

## 3 Common methods of measuring psychosocial stress

A stress response comprises diverse facets, including cognitions, emotions, behavior, and physiological symptoms. Depending on the research question, it is therefore possible to measure the emotions and cognitions underlying subjective stress via self-report or interviews, stress-associated behavior via behavioral codings in stressful situations, and physiological activation using markers of HPA axis and ANS activity. Typically, stress experience is also classified in terms of its duration, ranging from acute stress events via significant life events to chronic stress. In the following sections we shortly outline the most frequently employed measurement methods within each of these response levels, also touching on their limitations specifically in the context of studying the sequelae of quiet repression for a more detailed account, see [25,34].

### 3.1 Self-report measures

A widely used and cost-effective method of assessing psychosocial stress is via self-report. The most frequently employed self-report measures of chronic stress is the Perceived Stress Scale [35], which assesses the appraisal of life as stressful, defined as the extent to which an individual perceives that demands exceed their ability to cope. To gauge acute stress, one-item scales (e.g. “how stressed do you feel at this moment?“) or the state form of the State Trait Anxiety Inventory STAI [36] find wide application. Next to chronic and acute stress responses, there are also questionnaires and interviews to measure *life stress*, that is, the culmination of stressors experienced throughout the life course e.g., the Life Events Checklist [37].

There are several limitations to using self-report measures. Most importantly, they are prone to self-report bias [38,39]. This becomes apparent in the fact that subjective and hormonal stress measures often show little to no association “lack of psychoendocrine covariance” [40]. Additionally, definitions and expressions of “feeling stressed” may differ between individuals living in different societies and experiencing different social environments [25]. Lastly, concerning measures of stressor exposure, existing scales, such as the Life Event Checklist, are culturally dependent [25], and might not reflect all possible stressors of an individual's life.

### 3.2 Physiological measures

To measure the physiological stress response, ANS and HPA axis activity are most commonly assessed. Within the ANS, sympathetic activity is gauged by markers such as heart rate, blood pressure, or pupil dilation; parasympathetic activity by heart rate variability or respiratory sinus arrhythmia. Cortisol as the end hormone of the HPA axis can be sampled in blood, saliva, or hair. Among downstream markers of stress-related health, the pro-inflammatory cytokine interleukin-6 (IL-6) and the surrogate marker of low-grade inflammation, high sensitive C-reactive protein (hs-CRP), are indicative of inflammatory states [41]. Telomere length is associated with aging and age-related diseases [42,43].

### 3.3 Stress reactivity in the laboratory and in the field

The aim of inducing stress in the laboratory is to measure the stress response to a standardized acute challenge. Several laboratory stressors have been implemented, the most frequently used and most potent being the Trier Social Stress Test TSST [44] In the TSST, participants undergo a 5-min mock job interview, followed by a 5-min mental arithmetic task, in front of a mixed gender committee of two ‘behavioral analysts’ trained to maintain neutral nonverbal behavior, and to not engage with the target. This stress paradigm has been shown to reliably provoke subjective and physiological stress responses [45,46].

To assess stress in everyday life, ecological momentary assessment (EMA) studies have gained great popularity. Increasingly, data assessments are based on smartphones or other digital devices. Assessments of daily experience are frequently combined with the sampling of ANS activity and diurnal cortisol release e.g. Ref. [47].

### 3.4 Psychosocial stress in the context of political repression

To fully understand its severe and long-lasting impact, psychosocial stress experience in the shape of quiet repression needs to be evaluated in the historical and societal context of the former GDR. Unemployment, a possible implication of quiet repression, for example, was considered a criminal instance of “asocial behavior” (§249 StGB of the GDR). Next to existential fears, unemployment therefore triggered culture-specific identity threats which held distinct meanings in the socialist GDR compared to today's democratic Germany. Therefore, it is crucial not to simply evaluate stressors such as unemployment from one's own perspective, but rather to collect information (e.g., via interviews or historical/sociological sources) on what exactly constitutes a stressor in one's focus of research.

The fact that the GDR ceased to exist over 30 years ago offers a unique research opportunity in that there are few to no political barriers to obtaining information about state measures, as long as they are still available. Furthermore, a substantial population of individuals who grew up and were socialized in the GDR are still alive today. These victims urgently need to be heard in order to understand the extent of long-term suffering and debilitation inflicted by quiet repression measures.

## 4 Researching stress in the context of quiet political repression

We recently conducted a study on the psychobiological consequences of quiet political repression in the GDR, aiming to more closely understand health impairments in the victims. In the repression (experimental) group, participants were included if they were former GDR citizens who had experienced non-criminal repression in the GDR. This included, but was not limited to, having experienced at least two of the following state-organized repression techniques: Planned surveillance, summoning to interrogations, spreading of rumors or harassment at work/school. Participants of the control group were included if they were former GDR citizens who had not experienced any type of repression in the GDR or elsewhere. Experimental (n = 50) and control (n = 50) groups were demographically matched. Participant's age ranged between 50 and 80 years.

Exclusion criteria were (1) the experience of political imprisonment at any point in life, (2) using medication interfering with cortisol release (e.g. steroids, antidepressants), (3) active symptoms of affective disorders or PTSD in the last 2 months, (3) active symptoms of schizophrenia or any other psychotic disorder in the last 2 years, (4) excessive use of alcohol and/or other recreational drugs, and (5), only for those participants attending a psychosocial laboratory stressor (TSST; Kirschbaum et al., 1993), any severe physical health impairment that could be triggered in the acute stress setting (e.g. asthma, cardiovascular disease). Participants unable to attend the TSST for health-related reasons could nevertheless participate in the other study parts.

Repression experiences naturally occurred many years ago, and the psychophysiological makeup of our sample has undoubtedly been shaped by a multitude of influences since. Therefore, we opted to use a multimethod approach to best capture distinct facets of the stress response, including questionnaires and interviews, acute laboratory-based challenge tasks, and home sampling, spanning several physiological markers as indicators of the stress and immune systems. In the following, we provide a short summary of our study design, and then focus on the challenges encountered throughout the study process.

### 4.1 Study design

Participants were recruited via flyers, local newspaper announcements, and with the help of local institutions (e.g. churches, community colleges). Upon initial contact, a telephone interview was conducted to determine inclusion in experimental or control groups, or, else, exclusion from participation.

Individuals meeting inclusion criteria received a questionnaire (per choice either digitally or paper-pencil) to collect biographical data and information on individual perceptions of stress across the lifespan, as well as relevant related concepts, such as anxiety, depression, experience in close relationships, loneliness, and sleep quality. Repression victims additionally underwent a semi-structured interview focusing on GDR-related experiences, particularly the quiet repression measures they experienced, reasons for being targeted, feelings and thoughts related to their experiences as well as personal coping strategies, both then and now. Further, there was a section on how their lives have evolved after the end of the GDR in 1989/90. The interview was recorded and transcribed.

Following the interview, a third (second in the control group who did not attend the qualitative interview) session was scheduled for the collection of physiological data. Participants gave a blood sample to assess IL-6 and CRP as markers of the immune system, and telomere length as a marker of cell aging. They subsequently underwent the TSST. Throughout the TSST, participants provided a total of nine saliva samples for cortisol assessment, nine STAI state questionnaires, as well as 75 min of continuous ECG recoding. Finally, as part of a daily life ecological momentary assessment, participants collected seven saliva samples (again for cortisol assessment) and questionnaires on stress, sleep and mood throughout three separate days.

### 4.2 Challenges regarding the selection of inclusion and exclusion criteria

The exclusion criteria we set for the current study disqualified individuals with acute psychopathology, those using cortisol-affecting medications (e.g., antidepressants, corticosteroids), and, in the case of the TSST, individuals with severe physical impairments. We deemed these criteria essential for the interpretation of the stress and immune system markers, which would be difficult to read accurately if influenced by these external variables. Also, we wished to ensure the stability of participants, thus reducing the risk of triggering illness or retraumatization. However, as research indicates that quiet repression experiences can lead to psychopathology, we thereby excluded a considerable portion of our target population, introducing a significant bias to the final sample.

Our preliminary recruitment data reflect this stringent selection process. Of 168 initially interested individuals, 117 participated in a telephone screening. In the repression group, 63 individuals were screened of which 15 were excluded due to depression, anxiety, or medication use. For the TSST, an additional 19 individuals were excluded based on personal choice or physical illness. For the control group, 54 individuals were screened, with six exclusions and one additional exclusion for the TSST based on physical illness. These figures highlight the composition of our sample. Given that we are most likely examining a subpopulation – likely the most stable among the affected individuals – we expect that found differences would be even more pronounced in the general population. Therefore, when interpreting our study findings, it is essential to consider the composition of the final sample.

### 4.3 Specific challenges related to recruitment

Almost 35 years have passed since the collapse of the GDR. For several reasons, finding former GDR residents who were willing and eligible to participate in our study proved to be a significant challenge. Hence, the final sample has some inherent limitations that merit discussion.

#### 4.3.1 Willingness to participate

Contacting the target population proved to be a challenge in and on itself. Primarily, other than for individuals imprisoned during the GDR era, victims of quiet repression in the GDR are usually not organized in groups, thus complicating efforts for collective contacting. In consequence, we needed to search within the broader population to identify those who had experienced repression. Second, many affected individuals preferred to distance themselves from past events, leading to rejections in recruitment attempts. Third, affected individuals frequently believed they were alone in their experiences, and that their stories were of little interest to others. Strong self-doubt regarding their perceptions therefore inhibited them from sharing their ordeals, despite the enduring impact these experiences had on their lives. Nonetheless, once engaged, many participants expressed happiness and gratitude for the opportunity to share their stories. Last, and causing resistance to participate that was specific to the control group, some individuals were apprehensive that the study might discredit the GDR and undermine a significant aspect of their identity, an issue that still is of importance in general political discussions related to the transition in Germany.

#### 4.3.2 Eligibility and inherent bias of the repression sample

Some affected individuals, while grateful for the opportunity to process their experiences and partake in the study, did not meet inclusion criteria. As elaborated in more detail in section 4.2, reasons for this were, for example, dependency on psychotropic medication or having been subject to political imprisonment *in addition* to their repression experiences. Overall, any former victim of repression included in the study needed to have a certain level of stability and health – the exact factors compromised by the repression. This naturally led to the exclusion of the most severely affected individuals, and vice versa, an overrepresentation of those able to maintain a good level of functionality. Conversely, there is the possibility that someone would have experienced repression but not be compromised today. This subgroup of repression victims may not have been interested in study participation, leading to a sample likely not including both, the most and the least affected.

#### 4.3.3 Group allocation

We based group allocation on participant self-report rather than demanding objectifiable proof of repression measures (e.g. archival secret service records). This approach was chosen because for many victims of quiet repression such records never existed, or else, were already destroyed. Additionally, being doubted in their accounts of repression is a repeated and particularly painful experience for victims, the reactivation of which we wished to prevent in the context of our study. Therefore, extensive interviews were conducted to allow for a thorough and plausible understanding of the nuanced repression experiences. The GDR deliberately magnified the mythologization and a general sense of fear of the MfS by concealing information about its employees, structures, and areas of activity. As a result, the MfS gained an element of omnipresence in all areas of life in East German society, even if not targeting someone in particular [48]. Since the control group also encountered this structural repression, (self-)categorization as part of either the repression or the control group was not without challenge.

When relying on self-reports, whether through interviews or questionnaires, there is always the potential for memory bias. To mitigate this issue, we used historical sources to confirm whether the studied type of repression did, in fact, occur in the GDR, and to explain the measures that were used. This approach will allow to assess whether participants' reports align with historical sources. Ultimately, however, the story of one's life is always subjectively biased.

#### 4.3.4 Heterogeneity of repression measures

Because quiet repression was oftentimes tailored to the individual life situations and weaknesses of single victims, the recruited repression sample experienced extremely heterogeneous types and intensity of measures. Repression ranged from being repeatedly summoned to MfS interrogations without further restrictions, to being permanently destabilized by “disintegration measures,” which had fundamental effects on individuals’ intimate relationships, families, public reputation, life decisions, and ultimately entire biographies. However, attempting to distinctly operationalize or standardize repression measures would have inadequately captured the reality, since heterogeneity of measures is a fundamental aspect of quiet repression.

### 4.4 The role of qualitative interviews

Despite the high effort required for qualitative interviews, their execution held great importance for our study. For one thing, the individual narratives of each participant generated a wealth of detailed data that could not have been captured through questionnaires alone, and that will much enhance this understudied field. Furthermore, they provided a space for participants to share their personal life stories, which many had never done prior. Next to allowing for a new level of acceptance of their own experiences, participants emphasized the sense of significance they found in contributing to the scientific reprocessing of political repression, hoping to one day help other victims receive better support and understanding.

In addition to analyzing the wealth of interview material qualitatively, we plan on extracting categories from the interviews for each individual, which will describe the forms of experienced repression, the length of repression experience, the age at onset, and other relevant information. As of yet, we do not have specific questionnaires in this field to collect such additional information. We will then use the categories extracted from the interviews to explain differences in physiological reactions or to identify subgroups within our very heterogeneous repression group.

### 4.5 Challenges within the TSST

Typically, the TSST takes place in a bare room (except for the necessary technical equipment such as recording devices), and the committee members sitting at a desk and dressed in white lab coats. Participants are instructed to stand behind a microphone facing the committee members. We introduced several adjustments to this setup to ensure manageability of the TSST for the vulnerable repression sample. Thus, cameras and microphones were removed, white laboratory coats omitted, and the research assistants involved in testing underwent a training raising awareness for the sensitivities of the repression group, and providing guidelines on how to handle psychological emergency situations.

Despite these adjustments, it became apparent that the TSST was still too strong a stressor for some of the repression victims. Particularly, participants stated that the task surroundings (bare room, spatial constraints, lack of natural light, Covid-19 masks, testing room location in basement) were reminiscent of GDR State Security interrogation rooms, triggering a strong feeling of uncontrollability and bringing back long suppressed memories. To avoid retraumatization, stress testings were consequently shifted to a well-lit, spacious room, allowing participants to keep a “safety distance” from the TSST committee members. Additionally, to enhance a feeling of control, participants were informed of the general content of the TSST beforehand and committee members briefly introduced themselves before taking on their role in the task. All of these changes were implemented based on feedback of prior participants. With these alterations in place, participants continued to report considerable stress, but no feelings of retraumatization occurred.

### 4.6 Challenges with home sampling

The comprehensive ecological momentary assessment data collection presented challenges for our sample, particularly due to the advanced age of participants. Utilizing smartphones for digital data acquisition was often declined and generally susceptible to errors in technology usage. Although the alternative of analog data collection was often favored, participants' reliance on memory for measurement times also resulted in missing data. In summary, older individuals require additional support in navigating digital devices.

### 4.7 Challenges following data acquisition

Since study participation focused on potentially traumatic experiences, it reactivated long suppressed memories and negative feelings in many participants, which continued to resonate in the aftermath of the study. As a result, participants maintained ongoing contact with the study team, seeking reassurance through phone calls or follow-up meetings. Six participants required advisory or therapeutic care after participation underlining the need to provide psychological support in the background of such a study. Having contact information of self-help groups, topic-related counselling and specialized psychological practices readily available was therefore beneficial and reassuring for both participants and experimenters, and enabled to provide participants with the needed support and care. It is recommended to also provide emotional support for the interviewers who can be confronted with burdensome experiences and material. Additionally, as studies in this context are inherently political, public communication matters demand a high level of sensitivity.

## 5 Conclusion

Quiet repression is not a relic of the past, but still highly relevant today. Even if many people suffer from the consequences, in-depth knowledge of these forms of repression, their physiological mechanisms and health sequelae is scarce. Despite offering clear advantages, especially regarding to political barriers and access to information about state measures, investigating the consequences of past quiet repression is not without challenges: First, recruiting can be difficult due to victims’ age, health status, and willingness and ability to participate. Second, because of its inherent heterogeneity, delineating exact criteria for quiet repression and the affected target population is problematic. Third, to comprehensively capture the consequences of quiet repression, data collection methods need to be sensitively adapted to the special requirements of this population. Additionally, because the investigation may trigger emotional processing, psychological support and the possibility of referral to support networks for those affected are crucial. Naturally, the arising challenges differ when studying populations that are subject to current quiet repression.

In general, conducting research on an understudied phenomenon such as quiet political repression, and involving a sensitive population requires an open mind for direct participant input. Scientists engaging in the topic should be prepared to be available for exchange, even beyond data collection. If available, self-help organizations, specialized care and therapeutic offers are immensely helpful to initially reach out to the target population and to provide support in the aftermath of the study. Sharing experiences that have long been kept “undercover” may trigger significant emotional reactions in victims that cannot be processed in the research context. In conclusion, flexibility is essential to adapt to arising difficulties in the course of the study, and to provide an appropriate framework for a particular target group. This presents a particular challenge in understudied fields, as not all emerging problems can be anticipated in advance. Regarding quiet political repression, the current lack of knowledge only underscores the importance of delving deeper into this research field in the future.

## Author note

This article was not preregistered.

## Funding

R.M and B.S are part of the multicenter-project “10.13039/100018696Health consequences of 10.13039/100022835SED injustice” which receives funding from the German 10.13039/100021130Federal Ministry for Economic Affairs and Climate Action (Bundeshaushalt 2021, Kapitel 0910, Titel 68603).

## CRediT authorship contribution statement

**Ruth Marheinecke:** Writing – review & editing, Writing – original draft, Project administration, Investigation, Conceptualization. **Ann-Christin Winter:** Writing – original draft, Investigation, Conceptualization. **Bernhard Strauss:** Writing – review & editing, Funding acquisition. **Veronika Engert:** Writing – review & editing, Supervision, Conceptualization.

## Declaration of generative AI and AI-assisted technologies in the writing process

During the preparation of this work the first author used ChatGPT in order to streamline single sentences within the manuscript. After using this tool/service, all authors reviewed and edited the content as needed and take full responsibility for the content of the publication.

## Declaration of competing interest

No conflict.
